# Specimen self-collection for SARS-CoV-2 testing: Patient performance and preferences—Atlanta, Georgia, August-October 2020

**DOI:** 10.1371/journal.pone.0264085

**Published:** 2022-03-09

**Authors:** Kevin O’Laughlin, Catherine C. Espinosa, Sarah E. Smith-Jeffcoat, Mitsuki Koh, George M. Khalil, Adam Hoffman, Paulina A. Rebolledo, Marcos C. Schechter, Rebekah J. Stewart, Juliana da Silva, Caitlin Biedron, Bettina Bankamp, Jennifer Folster, Amy S. Gargis, Michael D. Bowen, Ashley Paulick, Yun F. Wang, Jacqueline E. Tate, Hannah L. Kirking, CDC Surge Diagnostic Testing Laboratory, CDC COVID-19 Emergency Response GA-10 Field Team

**Affiliations:** 1 COVID-19 Emergency Response, Centers for Disease Control and Prevention, Atlanta, GA, United States of America; 2 Epidemic Intelligence Service, CDC, Atlanta, GA, United States of America; 3 Grady Memorial Hospital, Atlanta, GA, United States of America; 4 Emory University Atlanta, Atlanta, GA, United States of America; Waseda University: Waseda Daigaku, JAPAN

## Abstract

Self-collected specimens can expand access to SARS-CoV-2 testing. At a large inner-city hospital 1,082 participants self-collected saliva and anterior nasal swab (ANS) samples before healthcare workers collected nasopharyngeal swab (NPS) samples on the same day. To characterize patient preferences for self-collection, this investigation explored ability, comfort, and ease of ANS and saliva self-collection for SARS-CoV-2 testing along with associated patient characteristics, including medical history and symptoms of COVID-19. With nearly all participants successfully submitting a specimen, favorable ratings from most participants (at least >79% in ease and comfort), and equivocal preference between saliva and ANS, self-collection is a viable SARS-CoV-2 testing option.

## Introduction

As the COVID-19 pandemic continues into a second year [1], accessible SARS-CoV-2 testing is important for resuming in-person activities such as medical care [2], travel [3], and school attendance [4]. Nasopharyngeal swab (NPS) testing has been the standard for SARS-CoV-2 detection, but non-nasopharyngeal specimens have shown similar sensitivity when compared to NPS [5–7]. With ongoing shortages of personal protective equipment (PPE) and swabbing materials, exploration of non-nasopharyngeal SARS-CoV-2 testing modalities, such as saliva, is critical for reducing health care worker exposures and the burden on healthcare resources [8–10]; self-collection of these specimens could further lessen that burden. Previous studies have demonstrated the public’s reported willingness to self-collect specimens [11], and the preference of nasal mid-turbinate or saliva collection compared to NPS [12, 13]. Limited data are available on the self-collection experience and preferences associated with demographics and symptoms. To further characterize patient preferences for self-collection, this investigation explored ability, and comfort and ease of anterior nasal swab (ANS) and saliva self-collection for SARS-CoV-2 along with associated patient characteristics, including medical history and symptoms of COVID-19.

## Methods

This analysis used data collected as part of a cross-sectional investigation comparing performance of self-collected saliva and ANS to healthcare worker-collected NPS on the same day from patients at Grady Memorial Hospital in Atlanta, GA, as previously described [14]. Briefly, patients were eligible if a SARS-CoV-2 reverse transcription-polymerase chain reaction (RT-PCR) test by NPS was ordered by the treating clinician. Patients were excluded if unable to consent, <18 years old, unable to self-collect both specimens, or an NPS was contraindicated. A trained interviewer used a standard questionnaire to collect participant demographics, medical history, and symptoms (S1 File). Medical conditions that may affect dexterity were defined as: arthritis (including osteoarthritis and unspecified type), multiple sclerosis, cerebral palsy, and carpal tunnel syndrome. Participants were able to add any other medical condition as ‘other’.

**Fig 1:**
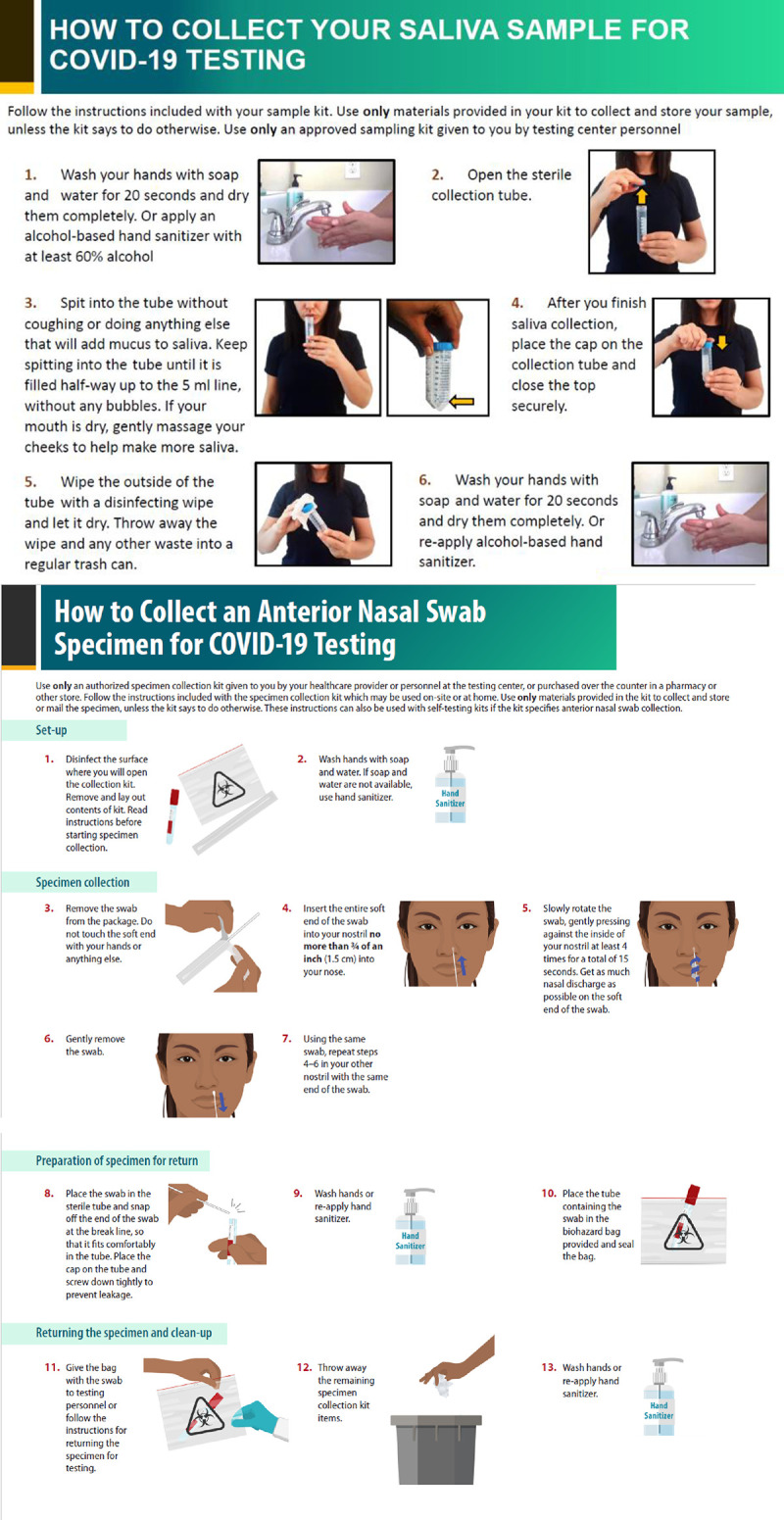
Saliva (A) and anterior nasal swab (B) self-collection instructional sheet handed to participants at Grady Memorial Hospital—Atlanta, GA, August–November 2020.

Participants self-collected saliva and ANS using standardized infographics for instructions (Fig 1A and 1B). Saliva was collected in a sterile 50 mL conical centrifuge tube. For ANS collection, participants inserted one mini flocked tip swab into both anterior nares and twirled for 15 seconds in each naris. Participants rated the collection comfort either positively (very comfortable or comfortable) or negatively (uncomfortable or very uncomfortable) and ease either positively (very easy or easy) or negatively (difficult or very difficult) for each self-collected specimen, and overall preference for specimen collection type. Successful self-collection was defined as a sample collected from a participant that was not rejected by the lab. An unfavorable rating was defined as at least one negative rating by a participant from the categories of very uncomfortable/uncomfortable or very difficult/difficult. After self-collection and interview, an NPS was collected by hospital clinicians. Participants were compensated with a $25 gift card. All specimens were tested at Centers for Disease Control and Prevention (CDC) using the CDC 2019-nCoV RT-PCR Diagnostic Panel [15]. All data were entered and stored in a REDCap database (v10.0.8, Vanderbilt University, Nashville, TN) hosted at CDC. SAS (version 9.4; SAS Institute) was used to conduct all analyses. Ease and comfort data were analyzed categorically. RT-PCR results of self-collected specimens were matched to NPS result to assess concordance. Chi-square tests were used to determine statistical significance of bivariate associations; an alpha of 0.05 was considered significant. T-tests were performed for continuous variables. This investigation was reviewed by CDC and conducted consistent with applicable federal law and CDC policy (See e.g., 45 C.F.R. part 46, 21 C.F.R. part 56; 42 U.S.C. 241(d); 5 U.S.C. 552a; 44 U.S.C. 3501 et seq.). It was determined to be an exempt public health activity by the Emory University Institutional Review Board and Grady Memorial Hospital Research Oversight Committee.

## Results

Among 1082 participants, 47.8% were female, mean age was 50 years (IQR 38–61), 81.1% were non-Hispanic Black, 80.9% reported an underlying medical condition associated with severe COVID-19 outcomes [15], and 18.1% reported concern for having COVID-19. Eighty percent (n = 866) were enrolled from the emergency department, 18.6% (n = 201) from the pre-operative screening clinic, and 1.4% (n = 15) from the Labor and Delivery department (Table 1). In the emergency department, 76.0% of participants reported at least one symptom consistent with COVID-19, compared to just 18.9% in the pre-procedure screening clinic. Concordance of RT-PCR results between self-collected specimens and healthcare-collected NPS was not associated with preference or ease/comfort ratings (S1 Data).

**Table 1:** Characteristics and reported symptoms of participants who contributed self-collected saliva and anterior nasal swabs for SARS-CoV-2 detection by RT-PCR at Grady Memorial Hospital—Atlanta, GA, August–October 2020.

Characteristic	Total
	n = 1082 n (%)
**Sex**	
Female	517 (47.8)
Male	560 (51.8)
Non-binary	2 (0.2)
Refused/Unknown/Missing	3 (0.3)
**Age group**	
18–29	147 (13.6)
30–44	223 (20.6)
45–59	404 (37.3)
60+	306 (28.3)
Missing	2 (0.2)
**Race/Ethnicity**	
American Indian/Alaska Native, non-Hispanic	2 (0.2)
Asian, non-Hispanic	10 (0.9)
Black, non-Hispanic	878 (81.1)
Hispanic/Latino	71 (6.6)
Multiple Races	16 (1.5)
Native Hawaiian/Pacific Islander, non-Hispanic	1 (0.1)
Unknown/Other/Refused	23 (2.1)
White, non-Hispanic	81 (7.5)
**Enrollment Location**	
Emergency department	866 (80.0)
Labor and delivery department	15 (1.4)
Preoperative screening clinic	201 (18.6)
**Reason for visit**	
Admission to Labor & Delivery Unit	10 (0.9)
COVID-19 concern	196 (18.1)
No COVID-19 concern	692 (64.0)
Preoperative requirements	181 (16.7)
Missing	3 (0.3)
**COVID-19 symptom status**	
Currently symptomatic	608 (56.2)
None reported	384 (35.5)
Symptomatic within 14 days	88 (8.1)
Missing	2 (0.2)
**Chronic medical conditions**	
No condition reported	272 (25.1)
At least one condition reported	810 (74.9)
At least one risk factor ^a^ for severe COVID-19 outcome	875 (80.9)
Emphysema/COPD ^b^	91 (8.5)
Asthma ^c^	226 (20.9)
Other chronic lung disease ^d^	65 (6.0)
Diabetes Mellitus (Type I or II)^e^	260 (24.0)
Hypertension (high blood pressure)^f^	493 (45.6)
Chronic heart or cardiovascular disease ^g^	155 (14.3)
Chronic kidney disease ^h^	76 (7.0)
Liver disease ^i^	40 (3.7)
Immunocompromising conditions ^j^^,^ ^k^	91 (8.4)
Neurologic/neurodevelopmental disorders ^l^^,^^m^	111 (10.3)
Cancer (including in remission)^n^	99 (9.1)
Other chronic diseases ^o^	178 (16.5)
Dexterity impairment ^p^	22 (2.0)
**Individual symptoms within 14 days** ^**q**^	
Fever, measured	110 (10.2)
Fever, subjective	247 (22.8)
Cough	434 (40.1)
Shortness of breath/difficulty breathing	448 (41.4)
Fatigue	416 (38.4)
Muscle or body aches	350 (32.3)
Headaches	348 (32.2)
New loss of taste	138 (12.8)
New loss of smell	98 (9.1)
Sore throat	209 (19.3)
Congestion/runny nose	331 (30.6)
Nausea	243 (22.5)
Vomiting	158 (14.6)
Diarrhea	232 (21.4)

^a^ Medical conditions identified by CDC that may put people at increased risk for severe outcomes from covid-19 and supported by meta-analysis/systemic review included cancer, neurological disease including cerebrovascular disease, chronic kidney disease, COPD (chronic obstructive pulmonary disease), diabetes mellitus (type 1 and type 2), heart conditions (such as heart failure, coronary artery disease, or cardiomyopathies), obesity (BMI ≥30 kg/m2), pregnancy, and smoking.

^b^ Emphysema status was missing (10) and unknown (10) for some participants

^c^ Asthma status was missing for 14 participants

^d^ Other chronic lung disease status was missing (4) and unknown (7) for some participants

^e^ 7 Diabetes Mellitus status was missing (4) and unknown (1) for some participants

^f^ Hypertension status was missing (5) and unknown (6) for some participants

^g^ Chronic cardiovascular disease status was missing (5) and unknown (14) for some participants

^h^ Chronic kidney disease status was missing (5) and unknown (10) for some participants

^I^ Liver disease status was missing (7) and unknown (8) for some participants

^j^ HIV co-infection (not virally suppressed), chemotherapy within past 12 months, solid-organ or bone marrow transplant, long-term steroid use (20 mg for >1 month), taking immunosuppressants, taking TNF-alpha inhibitors

^k^ Immunocompromising condition status was missing (7) and unknown (9) for some participants

^l^ Examples include seizure disorders such as epilepsy, Alzheimer’s, dementia, traumatic brain injuries, stroke/CVA

^m^ Neurologic/neurodevelopmental disorder status was missing (7) and unknown (6) for some participants

^n^ Cancer (including in remission) status was missing (84) and unknown (116) for some participants

^o^ Other chronic disease status was missing (27) and unknown (4) for some participants

^p^ Medical conditions that may affect dexterity were defined as: Arthritis (including osteoarthritis and unspecified type), multiple sclerosis, cerebral palsy, and carpal tunnel syndrome.

^q^ Symptom status was missing for 2 participants.

Overall, 48.5% of participants preferred saliva collection (over ANS), 42.7% preferred ANS (over saliva), and 4.5% had no preference between the two self-collected samples (Table 2). Saliva was preferred more often by those who were immunocompromised (64.4%, p = 0.03). In contrast, those with neurological disorders preferred ANS (55.8%, p = 0.05). Among 205 participants rating saliva self-collection unfavorably, more were experiencing symptoms compared to those rating saliva favorably (65.9% vs. 53.9%; p<0.01), specifically led by nausea (29.3% versus 20.8% p<0.01), cough (48.3% versus 38.4%, p<0.01), and shortness of breath (49.3% versus 39.4%, p<0.01). This group of 205 was also overrepresented in the emergency department (87.8% versus 78.1%, p<0.01) and had a higher proportion of reported history of lung disease (COPD/emphysema or asthma) (33.2% versus 23.7%; p<0.01) compared to those with favorable ratings. The 242 participants who rated ANS unfavorably reported nausea within 14 days of testing more often than those who rated collection favorably (27.7% versus 21.1%, p = 0.03); this group also reported a higher proportion of lung disease (31.4% versus 23.9%%; p = 0.02). Complete ease and comfort ratings are presented in Table 3. Most participants rated saliva and ANS self-collection positively (Saliva: 87.7% very easy/easy and 86.6% very comfortable/comfortable; ANS: 91.8% very easy/easy and 79.0% very comfortable/comfortable).

**Table 2:** Participant preference and favorability rating* for self-collected saliva and anterior nasal swabs by location, reported demographics, symptoms and underlying medical history, Grady Memorial Hospital, Atlanta, GA, August–October 2020.

	Preference				Ease/Comfort rating^b^					
	Saliva	ANS	None		Saliva			ANS		
					Favorable	Unfavorable		Favorable	Unfavorable	
Overall n = 1082 (100%)^a^	525 (48.5)	462 (42.7)	49 (4.5)		843 (80.4)	205 (19.6)		821 (77.2)	242 (22.8)	
Demographics	n (row %)			p-value	n (col %)		p-value	n (col %)		p-value
**Sex** ^**c**^										
Male	269 (50.3)	234 (43.7)	32 (6.0)	0.15	438 (52.1)	104 (50.7)	0.72	432 (52.7)	120 (49.8)	0.42
Female	255 (51.2)	226 (45.4)	17 (3.4)		402 (47.9)	101 (49.3)		387 (47.3)	121 (50.2)	
**Age group, years**										
18–29	82 (58.2)	52 (36.9)	7 (5.0)	0.06	113 (13.4)	30 (14.6)	0.58	113 (13.8)	31 (12.8)	0.94
30–44	117 (54.2)	95 (44.0)	4 (1.9)		181 (21.5)	37 (18.0)		171 (20.8)	50 (20.7)	
45–59	184 (46.9)	189 (48.2)	19 (4.8)		312 (37.0)	84 (41.0)		305 (37.1)	95 (39.3)	
60+	142 (49.5)	126 (43.9)	19 (6.6)		237 (28.1)	54 (26.3)		232 (28.3)	66 (27.3)	
**Race/Ethnicity**										
Black, non-Hispanic	436 (51.6)	368 (43.6)	41 (4.9)	0.15	694 (82.3)	157 (76.6)	0.18	668 (81.4)	196 (81.0)	0.92
Hispanic/Latino	36 (53.7)	26 (38.8)	5 (7.5)		54 (6.4)	16 (7.8)		52 (6.3)	18 (7.4)	
Other/Refused/Unk^d^	17 (36.2)	29 (61.7)	1 (2.1)		39 (4.6)	10 (4.9)		39 (4.8)	10 (4.1)	
White, non-Hispanic	36 (46.8)	39 (50.6)	2 (2.6)		56 (6.6)	22 (10.7)		62 (7.6)	18 (7.4)	
**Symptoms**										0.69
None	178 (49.2)	162 (44.8)	22 (6.1)	0.23	316 (37.5)	57 (27.8)	**<0.01**	292 (35.6)	84 (34.7)	
Any symptom within 14 days	41 (47.1)	45(51.7)	1 (1.1)		73 (8.7)	13 (6.3)		70 (8.5)	17 (7.0)	
Any current symptom	306 (52.1)	255 (43.4)	26 (4.4)		454 (53.9)	135 (65.9)		459 (55.9)	141 (58.3)	
Nausea within 14 days										
Yes	125 (53.2)	98 (41.7)	12 (5.1)	0.59	175 (20.8)	60 (29.3)	**<0.01**	173 (21.1)	67 (27.7)	**0.03**
No	400 (49.9)	364 (45.4)	37 (4.6)		668 (79.2)	145 (70.7)		648 (78.9)	175 (72.3)	
Cough within 14 days										
Yes	217 (51.5)	186 (44.2)	18 (4.3)	0.80	324 (38.4)	99 (48.3)	**<0.01**	320 (39.0)	110 (45.5)	0.07
No	308 (50.0)	276 (44.9)	31 (5.0)		519 (61.6)	106 (51.7)		501 (61.0)	132 (54.5)	
Shortness of breath within 14 days										
Yes	224 (52.2)	190 (44.3)	15 (3.5)	0.26	332 (39.4)	101 (49.3)	**<0.01**	331 (40.3)	109 (45.0)	0.19
No	301 (49.6)	272 (44.8)	34 (5.6)		511 (60.6)	104 (50.7)		490 (59.7)	133 (55.0)	
**Medical history**										
Lung disease ^e^										
Yes	136 (50.9)	121 (45.3)	10 (3.7)	0.68	200 (23.7)	68 (33.2)	**<0.01**	196 (23.9)	76 (31.4)	**0.02**
No	389 (50.6)	341 (44.3)	39 (5.1)		643 (76.3)	137 (66.8)		625 (76.1)	166 (68.6)	
CKD^f^										
Yes	32 (45.7)	37 (52.9)	1 (1.4)	0.20	53 (6.4)	20 (9.9)	0.08	58 (7.2)	17 (7.1)	0.97
No	489 (51.1)	421 (44.0)	47 (4.9)		781 (93.6)	183 (90.1)		753 (92.8)	223 (92.9)	
Immunocompromised^g^										
Yes	56 (64.4)	29 (33.3)	2 (2.3)	**0.03**	66 (7.9)	22 (11.1)	0.15	63 (7.7)	26 (11.0)	0.11
No	464 (49.5)	426 (45.5)	47 (5.0)		771 (92.1)	177 (88.9)		752 (92.3)	210 (89.0)	
Neurological d/o^h^^,^ ^i^										
Yes	42 (40.4)	58 (55.8)	4 (3.8)	**0.05**	83 (9.9)	22 (10.9)	0.68	85 (10.4)	22 (9.2)	0.58
No	481 (52.0)	399 (43.1)	45 (4.9)		754 (90.1)	180 (89.1)		730 (89.6)	217 (90.8)	
Dexterity Impairment^j^										
Yes	7 (35.0)	12 (60.0)	1 (5.0)	0.35	16 (1.9)	5 (2.4)	0.62	18 (2.2)	3 (1.2)	0.35
No	518 (51.0)	450 (44.3)	48 (4.7)		827 (98.1)	200 (97.6)		803 (97.8)	239 (98.8)	
**Location**										
Emergency Department (n = 866)	420 (50.6)	375 (45.2)	35 (4.2)	0.48	658 (78.1)	180 (87.8)	**<0.01**	651 (79.3)	198 (81.8)	0.54
Pre-op Screening Clinic (n = 201)	100 (51.8)	80 (41.5)	13 (6.7)		170 (20.2)	25 (12.2)		157 (19.1)	42 (17.4)	
Labor & Delivery (n = 15)	5 (38.5)	7 (53.8)	1 (7.7)		15 (1.8)	0		13 (1.6)	2 (0.8)	

**Abbreviations:** ANS = Anterior nasal swab; Unk = Unknown; COPD = Chronic obstructive pulmonary disease; CKD = Chronic kidney disease; D/o = disorder; Pre-op = Pre-operative.

^a^ There was a total of 1,036 participants who indicated a preference (46 missing), 1,048 participants who rated saliva collection (34 missing), and 1,063 participants who rated ANS collection (19 missing).

^b^ An unfavorable rating was defined as at least one negative rating by a participant from the categories of very uncomfortable/uncomfortable or very difficult/difficult.

^c^ Sex data missing from 3 participants who indicated a preference, and 3 participants who provide a saliva and ANS favorability ratings.

^d^ This category includes persons of all other races, those who did not provide race/ethnicity data, and those for whom race/ethnicity data are otherwise unavailable.

^e^ Lung disease includes COPD/emphysema and asthma.

^f^ CKD status missing from 9 participants who indicated a preference, 11 participants who provided a saliva favorability rating, and 2 participants who provided an ANS favorability rating.

^g^ Immunocompromised status missing for 12 participants who indicated a preference, 6 participants who provided saliva and ANS favorability ratings.

^h^ Neurological d/o was defined as any self-reported neurological disorder, including seizure d/o such as epilepsy, Alzheimer’s, dementia, traumatic brain injury, and stroke/CVA.

^I^ Neurological status was missing from 7 participants who indicted a preference, 6 participants who provided a saliva favorability rating, and 3 participants who provided an ANS favorability rating.

^j^ Medical conditions that may affect dexterity were defined as: Arthritis (including osteoarthritis and unspecified type), multiple sclerosis, cerebral palsy, and carpal tunnel syndrome.

**Table 3:** Ease and comfort ratings of participants who contributed saliva and anterior nasal swabs for SARS-CoV-2 detection by RT-PCR at Grady Memorial Hospital—Atlanta, GA, August–October 2020.

Rating	Ease and comfort ratings	
	Saliva	ANS*
	n (%)	
**Ease**	n = 1048	n = 1062
**Positive**	**919 (87.7)**	**975 (91.8)**
Very easy	411 (39.2)	414(39.0)
Easy	508 (48.5)	561(52.8)
**Negative**	**129 (12.3**)	**87 (8.2)**
Difficult	108 (10.3)	73 (6.9)
Very difficult	21 (2.0)	14 (1.3)
**Comfort**	n = 1044	n = 1060
**Positive**	**904 (86.6)**	**838 (79.0)**
Very comfortable	283 (27.1)	222 (20.9)
Comfortable	621 (59.5)	616 (58.1)
**Negative**	**140 (13.4)**	**222 (20.9)**
Uncomfortable	123(11.8)	193 (18.2)
Very uncomfortable	17 (1.6)	29 (2.7)

*ANS = Anterior nasal swab.

Nearly all participants were able to provide a sufficient saliva (95.5%) and ANS (99.4%) specimen for testing (Table 4). Sixteen (1.5%) participants were unable to collect saliva and three (0.3%) were unable to provide ANS samples. An additional 29 saliva samples were rejected for: improper storage (n = 27), and leakage (n = 2). Two ANS samples were rejected for improper storage. Thirty saliva samples (2.8%) had insufficient volume for testing; these participants were more often older (mean age 56.3 years versus 49.5 years, p = 0.02), women (63.3% versus 47.5%, p = 0.09), and self-reported having chronic kidney disease (16.7% versus 6.9%, p = 0.04) than their counterparts (Table 5). They did not have a higher proportion of dexterity impairment (0% versus 2.1%, p = 0.42) or neurological disorders (14.3% versus 10.2%, p = 0.48). Stratification of the 60+ age group did not show significant differences in preference, ratings, or ability (Supplemental data).

**Table 4:** Participant performance of ability to self-collect saliva and anterior nasal swabs for SARS-CoV-2 detection by RT-PCR at Grady Memorial Hospital—Atlanta, GA, August–October 2020.

	Self-collection	
	Saliva	ANS
	n = 1082 (%)	
**Collected**	1063 (98.2)	1077 (99.5)
**Declined**	3 (0.3)	2 (0.2)
**Unable**	16 (1.5)	3 (0.3)
Rejected for insufficient* **volume**	30 (2.8)	-
**Rejected** ^†^	29 (2.7)	2 (0.2)
**Successful self-collection**	1033 (95.5)	1075 (99.4)

*Insufficient volume was defined as less than 200 μL.

^†^ 29 saliva samples were rejected for: improper storage (n = 27), and leakage (n = 2). Two ANS samples were rejected for improper storage.

**Table 5:** Characteristics of participants unable to provide sufficient volume of self-collected saliva for SARS-CoV-2 detection by RT-PCR at Grady Memorial Hospital—Atlanta, GA, August–October 2020.

Demographics	Sufficient^a^ saliva volume submitted		
	Yes	No	p-value
	n (%)	n (%)	
**Overall**	1033 (97.2)	30 (2.8)	
**Sex** ^**b**^			
Male	540 (52.5)	11 (36.7)	0.09
Female	488 (47.5)	19 (63.3)	
**Mean Age**	49.5	56.3	**0.02**
**Symptoms** ^**c**^			
No	368 (35.7)	11 (36.7)	0.96
Symptoms within 14 days	84 (8.2)	2 (6.7)	
Current symptoms	579 (56.2)	17 (56.7)	
Nausea within 14 days	227 (22.0)	12 (40.0)	**0.02**
**Medical history**			
Dexterity impairment^d^			
Yes	22 (2.1)	0	0.42
No	1011 (97.9)	30	
Neurological disorder^e^^,^ ^f^			
Yes	104 (10.2)	4 (14.3)	0.48
No	918 (89.8)	24 (85.7)	
Chronic kidney disease^g^			
Yes	70 (6.9)	5 (16.7)	0.04
No	949 (93.1)	25 (83.3)	

^a^ Sufficient volume was defined as an adequate volume of saliva to allow the laboratory to properly run the SARS-CoV-2 diagnostic assay, approximately more than 200 μL.

^b^ Missing data on sex for 5 participants.

^c^ Missing data on symptoms for 2 participants.

^d^ Neurological d/o was defined as any self-reported neurological disorder, including seizure d/o such as epilepsy, Alzheimer’s, dementia, traumatic brain injury, and stroke/CVA.

^f^Medical conditions that may affect dexterity were defined as: Arthritis (including osteoarthritis and unspecified type), multiple sclerosis, cerebral palsy, and carpal tunnel syndrome.

^f^ Missing data on neurological disorders for 13 participants

^g^ Missing data on chronic kidney disease for 14 participants.

## Discussion

This investigation found that self-collected specimens for SARS-CoV-2 testing were an acceptable option to participants; nearly all were able to provide self-collected saliva and ANS specimens and rated the experience favorably in at least one category. High collection success rates indicate that no person should be automatically excluded from public health self-collection programs based solely on demographic factors. Those with potential dexterity impairment, including neurological disorders or arthritis, were just as likely to have success with self-collection. Importantly for reaching those disproportionally affected by the COVID-19 pandemic, this investigation was conducted in a public hospital and included many participants at increased risk for severe COVID-19 disease [16–18].

Since instructions for self-collection were given in written infographic format and did not rely on interviewers or healthcare personnel for teaching, self-collection testing programs need not be limited to healthcare settings. Self-collection testing could therefore potentially preserve valuable healthcare resources, including facilities, personnel, and PPE. Additionally, higher favorable ratings for both self-collected specimen types in asymptomatic individuals and those in pre-procedure screening suggest that self-collected specimens might be even more favorable in screening programs compared to clinical, diagnostic testing situations. Notably, although our study used SARS-CoV-2 RT-PCR testing, ANS are included as approved specimens for several SARS-CoV-2 antigen tests with Emergency Use Authorizations approved by the US Food and Drug Administration. The ability to screen with self-collected rapid antigen tests is an important tool for expanding access to testing, especially for situations where frequent testing has been proposed, such as in school settings [19, 20]. Self-tests are also now part of CDC’s risk reduction strategy along with other mitigation efforts [21]. Further studies can explore specifics to this approach by investigating modality preferences and the reasons behind those preferences for particular demographics, underlying medical conditions, or settings.

The very small subset of participants with difficulty providing saliva were older and possibly in poorer health; they were however able to provide ANS specimens, so offering both modalities in a testing program for this population, such as in a nursing home, might increase successful participation. Saliva was the preferred method of self-collection by a small margin; those with neurological disorders were the only group with a statistically significant preference for ANS. When attempting to broaden access with a self-collected specimen diagnostic program, either self-collection modality would likely be similarly acceptable in many settings, given the almost equal preference between the two.

This investigation has several limitations. First, despite written infographics being the main source of instruction, interviewers were present and an element of bedside manner or outside coaching may have contributed to ratings and preferences; therefore the experience may not translate from this element of interviewer presence to true unsupervised self-collection. Second, language translation services and Spanish instructional handouts were used, but communication difficulties (i.e., vision/hearing impairment, limited English proficiency) were not assessed; these may have affected understanding of the instructional handout despite being mostly pictorial. Third, 50 milliliter conical centrifuge tubes were used for saliva collection. These have a narrow mouth, which may have affected saliva-collection ratings. Fourth, the diagnostic performance report of this same investigation demonstrated that the self-collected saliva and ANS specimens had overall lower sensitivity than NPS specimens (saliva and ANS had sensitivities of 68% (95%CI: 55–78%), and 59% (95%CI: 47–70%), respectively) [14], which may limit application. The source of sensitivity limitations is discussed extensively in the aforementioned report. Fifth, all medical histories and symptoms were self-reported and may be subject to recall bias. Sixth, the categorical scale used to assess comfort and ease may have limited the ability to determine small differences between the two testing modalities. Future investigations that collect explanations of chosen ratings could help to explore why certain participants favored one specimen over another. Last, most participants were recruited from an emergency department in a large urban area and may not be representative of other communities in the United States.

Accessible SARS-CoV-2 diagnostic testing is important for routine patient care and resumption of in-person activities, and self-collected specimens can help expand access while reducing personnel and resource burdens on the healthcare system.

## Supporting information

S1 File Survey instrument for data collection at Grady Memorial Hospital—Atlanta, GA, August–November 2020. (PDF)

S1 Data Dataset for data collection at Grady Memorial Hospital—Atlanta, GA, August–November 2020. (XLSX)
