# Supplementary material for: Specimen self-collection for SARS-CoV-2 testing: Patient performance and preferences—Atlanta, Georgia, August-October 2020
Source: PLoS One. 2022 Mar 9;17(3):e0264085. doi: 10.1371/journal.pone.0264085 (PMC8906601; doi:10.1371/journal.pone.0264085)
Supplement: S1 File — (PDF) [file pone.0264085.s001.pdf]

DASH

2020-Project-Epi Questionnaire

Interviewer Information

Date of interview: ______/______/_______ (MM/DD/YYYY)

Enrollment Location: ☐ Grady ED ☐ Purple Pod ☐ Labor & Delivery ☐ Other (specify)___________________

Study ID (Official *use only*): __________________________

Interviewer: First Name: ______________________________ Last Name:__________________________

Participant Information

1. Age (years): ____________
2. Ethnicity: (Do you identify as Hispanic or Latino?) ☐ Hispanic/Latino ☐ Non-Hispanic/Latino ☐ Not specified ☐ Refused
3. Race: (check all that apply) ☐ White ☐ Asian ☐ American Indian/Alaska Native ☐ Black ☐ Native Hawaiian/Other Pacific Islander ☐ Unknown ☐ Refused ☐ Other_________________
4. Sex: ☐ Male ☐ Female ☐ Non-binary ☐ Refused

Symptoms, Clinical Course, Treatment, Testing, and Outcome

5. Is COVID-19 the reason that brought you to the hospital today? ☐ Yes, COVID-19 concern ☐ No, no COVID-19 concern ☐ Pre-operative Requirements ☐ Admission to Labor & Delivery Unit

   A. If not COVID-19, what concern brought you to the hospital/clinic today? ____________________________________

6. I'm going to read a short list of symptoms. If I name one you've had in the last 14 days, please stop me and let me know. Symptoms of coronavirus may include fever or chills, cough, shortness of breath or difficulty breathing, fatigue, muscle or body aches, headache, new loss of taste or smell, sore throat, congestion or runny nose, nausea or vomiting, and diarrhea.

   ☐ Yes ☐ No ☐ Unknown

   IF NO OR UNKNOWN, SKIP to Q7. IF YES, CONTINUE TO Q6A.

   A. If yes, what date did your first symptom associated with this illness start? _______/_______/_________ (MM/DD/YYYY) ☐ Unknown. If date cannot be remembered, record month when illness started _______________________.

   B. I'm going to read a short list of symptoms you may have experienced in the last 14 days. You may respond "Yes", "No", or "I don't know" to whether you've had any of these in the last 14 days. I will also ask if you are <u>currently</u> experiencing any of these symptoms.

Symptom	Symptoms in the past 14 days	Current symptoms
Fever Measured by Thermometer (highest temp _________ ºF)	☐ Yes ☐ No ☐ Unknown	☐ Yes
Subjective fever (felt feverish)	☐ Yes ☐ No ☐ Unknown	☐ Yes
Cough	☐ Yes ☐ No ☐ Unknown	☐ Yes
Shortness of Breath/Difficulty breathing	☐ Yes ☐ No ☐ Unknown	☐ Yes
Fatigue	☐ Yes ☐ No ☐ Unknown	☐ Yes
Muscle or body aches	☐ Yes ☐ No ☐ Unknown	☐ Yes
Headaches	☐ Yes ☐ No ☐ Unknown	☐ Yes
New loss of taste	☐ Yes ☐ No ☐ Unknown	☐ Yes
New loss of smell	☐ Yes ☐ No ☐ Unknown	☐ Yes
Sore throat	☐ Yes ☐ No ☐ Unknown	☐ Yes
Congestion/Runny Nose	☐ Yes ☐ No ☐ Unknown	☐ Yes
Nausea	☐ Yes ☐ No ☐ Unknown	☐ Yes
Vomiting	☐ Yes ☐ No ☐ Unknown	☐ Yes
Diarrhea	☐ Yes ☐ No ☐ Unknown	☐ Yes
Other, specify below:	☐ Yes ☐ No ☐ Unknown	☐ Yes

Other symptoms:____

2020-Project-Epi Questionnaire

Study ID: ________________

7. Since January 1st 2020, have you had any other illnesses that you think could have been COVID-19? ☐ Yes ☐ No ☐ Unknown
IF NO OR UNK, SKIP TO Q8. IF YES, CONTINUE TO Q7A-7B.

A. If yes, what date did the symptoms associated with this illness start? _____/_____/_____ (MM/DD/YYYY)
☐ Unknown If date cannot be remembered, record month when illness started ______________________.

B. I'm going to read a short list of symptoms you may have experienced during this illness. Respond "Yes", "No", or "I don't know" to each:

Symptom	Symptom Reported?	Symptom	Symptom Reported?
Fever measured by a thermometer (highest temp _________ °F)	☐ Yes ☐ No ☐ Unknown	New loss of smell	☐ Yes ☐ No ☐ Unknown
Subjective fever (felt feverish)	☐ Yes ☐ No ☐ Unknown	New loss of taste	☐ Yes ☐ No ☐ Unknown
Cough	☐ Yes ☐ No ☐ Unknown	Sore throat	☐ Yes ☐ No ☐ Unknown
Difficulty breathing	☐ Yes ☐ No ☐ Unknown	Congestion/runny nose	☐ Yes ☐ No ☐ Unknown
Fatigue	☐ Yes ☐ No ☐ Unknown	Nausea	☐ Yes ☐ No ☐ Unknown
Muscle or body aches	☐ Yes ☐ No ☐ Unknown	Vomiting	☐ Yes ☐ No ☐ Unknown
Headaches	☐ Yes ☐ No ☐ Unknown	Diarrhea	☐ Yes ☐ No ☐ Unknown
		Other, specify below:	☐ Yes ☐ No ☐ Unknown

Other:____

Medical History -- Past Medical History

8. Have you been tested for COVID-19 in the past? ☐ Yes ☐ No ☐ Unknown
IF NO OR UNKNOWN, SKIP TO Q9. IF YES, CONTINUE TO 8A-8B.

A. Did you have a swab of your nose or throat collected? ☐ Yes ☐ No ☐ Unknown
IF NO OR UNKNOWN, SKIP TO 8B.

A1. If yes, when was the swab taken? _______/_______/_______ (MM/DD/YYYY) ☐ Unknown
If date cannot be remembered, record month when illness started _________________

A2. What was the result of the swab test? ☐ Positive ☐ Negative ☐ Unknown

B. Did you have a blood sample for COVID-19 collected? ☐ Yes ☐ No ☐ Unknown
IF NO OR UNKNOWN, SKIP TO Q9.

B1. If yes, when was the blood sample taken? _______/_______/_______ (MM/DD/YYYY) ☐ Unknown
If date cannot be remembered, record month when illness started: _________________

B2. What was the result of the blood test? ☐ Positive ☐ Negative ☐ Unknown

9. Do you have any of the following chronic medical conditions? Please specify **ALL** conditions that qualify.

Condition	Response		
Emphysema/COPD	☐Yes	☐No	☐Unknown
Asthma	☐Yes	☐No	☐Unknown
Other chronic lung disease	☐Yes	☐No	☐Unknown
Diabetes Mellitus (Type I or II)	☐Yes	☐No	☐Unknown
Hypertension (High blood pressure)	☐Yes	☐No	☐Unknown
Chronic heart or cardiovascular disease	☐Yes	☐No	☐Unknown
Chronic kidney disease	☐Yes	☐No	☐Unknown
Liver disease	☐Yes	☐No	☐Unknown
Condition affecting your immune system or making it hard for you to fight infections*	☐Yes	☐No	☐Unknown
Neurologic/neurodevelopmental disorders or condition affecting your brain**	☐Yes	☐No	☐Unknown
Cancer (including in remission)	☐Yes	☐No	☐Unknown
Other chronic diseases	☐Yes	☐No	☐Unknown

If OTHER = YES, fill in here:____

**HIV co-infection (not virally suppressed), chemotherapy within past 12 months, solid-organ or bone marrow transplant, long-term steroid use (20 mg for >1 month), taking immunosuppressants, taking TNF-alpha inhibitors*

***Examples include seizure disorders such as epilepsy, Alzheimer's, dementia, traumatic brain injuries, stroke/CVA.*

10. How tall are you? Choose one: _____ft _____ (in) OR _________ (cm)

11. How much do you weigh? Choose one: __________ (lbs) OR _________ (kg)

12. Do you currently smoke cigarettes, cigars, or a pipe? ☐ Yes ☐ No ☐ Unknown

13. Do you currently vape tobacco or use e-cigarettes? ☐ Yes ☐ No ☐ Unknown

IF PATIENT IS FEMALE AND BETWEEN AGES 15-45 YEARS OLD:

14. Are you currently pregnant?
☐ Yes (weeks pregnant) ___________ ☐ No ☐ Unknown

Exposure Risk Factors—COVID-19

15. What best describes your housing situation in the past 14 days?
☐ House or apartment for just you or you and people in your family
☐ House or apartment you share with people not in your family
☐ Moving from house-to-house (i.e., couch surfing)
☐ Shelter
☐ No housing (i.e., tent or sleeping outside)
☐ Other_____________
☐ Prefer not to say

16. Have you ever had close contact with someone who had a positive COVID-19 test? (*If multiple exposures, record only the most recent exposure.*)
☐ Yes ☐ No ☐ Unknown
IF NO OR UNKNOWN, SKIP TO Q17. IF YES, CONTINUE WITH 16A-B.

A. When did that close contact occur? _____/_____/_____ (MM/DD/YYYY) (If over multiple days, please provide the first day of exposure)
☐ Unknown. If date cannot be remembered, record month when contact occured ______________________.

B. What was your relationship to the person with COVID-19? (Choose the most appropriate)

☐ HH member ☐ Family member, non-HH ☐ Friend ☐ Coworker ☐ Person encountered at work (not coworker)
☐ Person encountered in a healthcare setting (not coworker) ☐ Boy/girlfriend or significant other ☐ Other – specify_______________

Exposure Risk Factors—Employment

17. Are you currently employed, or have you done any work at any point in the last 14 days? ☐ Yes ☐ No

IF NO, SKIP to Q18. IF YES, CONTINUE WITH Q17 A-F.

A. What kind of work do you do? ____

B. What best describes your current (or former) place of employment?

☐ Healthcare facility ☐ Government/public service ☐ Grocery store/Gas station ☐ Restaurant/Bar ☐ Retail
☐ Cleaning service ☐ Construction/landscaping service ☐ Factory ☐ Delivery services (e.g., USPS, Fedex, Instacart, Uber Eats)
☐ Other ____

C. If working in the past 14 days, have you been:

☐ Working from home 100% ☐ Working outside home ☐ Mix of working inside/outside home

IF WORKING FROM HOME 100%, SKIP TO Q18. IF ANY WORK OUTSIDE HOME, CONTINUE to Q17 D-F.

D. If working outside your home, is your job ☐ Primarily indoors ☐ Primarily outdoors ☐ Mix of outdoors/indoors

E. If working outside your home, on average, how many people (i.e. co-workers, clients) do you have close contact with each hour while at work?

☐ <10 ☐ 10-20 ☐ >20

F. If working outside home, how often do you wear a mask while at work? ☐ Always ☐ Most of the time ☐ Sometimes ☐ Never

Exposure Risk Factors—Social

18. In the past 14 days, when you leave your home and you go inside another building like a grocery store, how often do you wear a mask?

☐ Always ☐ Most of the time ☐ Sometimes ☐ Never

19. In the past 14 days, did you participate in any of the following activities?

IF YES: Was the activity **indoors, outdoors, or both**?

	No	Yes	
Worship service, including church, mosque, temple, or other	☐	☐ Indoors	☐ Outdoors
Funeral	☐	☐ Indoors	☐ Outdoors
Choir practice	☐	☐ Indoors	☐ Outdoors
Rally or protest	☐	☐ Indoors	☐ Outdoors
Wedding	☐	☐ Indoors	☐ Outdoors
Exercise class	☐	☐ Indoors	☐ Outdoors
Sports practice	☐	☐ Indoors	☐ Outdoors
Sporting event	☐	☐ Indoors	☐ Outdoors
Other event with >10 people	☐	☐ Indoors	☐ Outdoors

2020-Project-Epi Questionnaire

Study ID: ________________

20. In the past 14 days, which of the following **activities** have you done?

	No	Yes
Grocery shopping	☐	☐
Any shopping other than groceries	☐	☐
Eating/drinking at indoor restaurant	☐	☐
Used public transportation	☐	☐
Traveled on a plane	☐	☐
Visited friends/family inside their home	☐	☐
Visited a healthcare facility (not including today's visit)	☐	☐

Specimen Collection Assessment

Think back to the saliva and the nose swab we just collected.

How comfortable was it for you collecting your own saliva (spit) and nasal (nose) sample?

	Very comfortable	Comfortable	Uncomfortable	Very uncomfortable	NA
21. Saliva (spit)	☐	☐	☐	☐	☐
22. Nasal (nose)	☐	☐	☐	☐	☐

How easy was it for you to collect your own saliva (spit) and nasal (nose) sample?

	Very easy	Easy	Difficult	Very difficult	NA
23. Saliva (spit)	☐	☐	☐	☐	☐
24. Nasal (nose)	☐	☐	☐	☐	☐

25. If you were able to choose to provide only one specimen, which one would you prefer to provide?
☐ Nasal ☐ Saliva ☐ NP Swab ☐ No Preference ☐ Not Applicable ☐ Refused

IF ONLY ONE SAMPLE WAS COLLECTED, PLEASE SELECT "NOT APPLICABLE".

That was my last question for you.
Thank you very much for taking the time to answer these questions.
This information is going to help us determine how to improve COVID-19 testing for the community.
Do you have any final questions for me?

END SURVEY

FOR COMPLETION BY OFFICIAL:

Specimens Collected

26. Self-collected nasal swab: ☐ Collected ☐ Not collected, patient declined ☐ Not collected, patient unable
27. Self-collected saliva: ☐ Collected ☐ Not collected, patient declined ☐ Not collected, patient unable
